# Parallel Helix Actuators for Soft Robotic Applications

**DOI:** 10.3389/frobt.2020.00119

**Published:** 2020-09-30

**Authors:** James H. Chandler, Manish Chauhan, Nicolo Garbin, Keith L. Obstein, Pietro Valdastri

**Affiliations:** ^1^Science and Technology of Robotics in Medicine (STORM) Laboratory, School of Electronics and Electrical Engineering, University of Leeds, Leeds, United Kingdom; ^2^Science and Technology of Robotics in Medicine (STORM) Laboratory, Department of Mechanical Engineering, Vanderbilt University, Nashville, TN, United States; ^3^Division of Gastroenterology, Hepatology, and Nutrition, Vanderbilt University Medical Center, Nashville, TN, United States

**Keywords:** soft robotics, soft materials, bending actuators, robot fabrication, kinematic model, soft robot applications, monolithic actuators, inflatable actuators

## Abstract

Fabrication of soft pneumatic bending actuators typically involves multiple steps to accommodate the formation of complex internal geometry and the alignment and bonding between soft and inextensible materials. The complexity of these processes intensifies when applied to multi-chamber and small-scale (~10 mm diameter) designs, resulting in poor repeatability. Designs regularly rely on combining multiple prefabricated single chamber actuators or are limited to simple (fixed cross-section) internal chamber geometry, which can result in excessive ballooning and reduced bending efficiency, compelling the addition of constraining materials. In this work, we address existing limitations by presenting a single material molding technique that uses parallel cores with helical features. We demonstrate that through specific orientation and alignment of these internal structures, small diameter actuators may be fabricated with complex internal geometry in a single material—without- additional design-critical steps. The helix design produces wall profiles that restrict radial expansion while allowing compact designs through chamber interlocking, and simplified demolding. We present and evaluate three-chambered designs with varied helical features, demonstrating appreciable bending angles (>180°), three-dimensional workspace coverage, and three-times bodyweight carrying capability. Through application and validation of the constant curvature assumption, forward kinematic models are presented for the actuator and calibrated to account for chamber-specific bending characteristics, resulting in a mean model tip error of 4.1 mm. This simple and inexpensive fabrication technique has potential to be scaled in size and chamber numbers, allowing for application-specific designs for soft, high-mobility actuators especially for surgical, or locomotion applications.

## Introduction

The compliant nature and large range of motion of soft robotic fluidic actuators engenders a wide application scope with significant research interest (Rus and Tolley, 2015; Laschi et al., 2016; Gorissen et al., 2017; Shintake et al., 2018; Chen et al., 2019; Gifari et al., 2019; Runciman et al., 2019). Actuator designs typically comprise one or more elastomeric materials with the optional addition of strain limiting material, with single and multi-chamber configurations being selected based on application requirements. Desirable motions, such as extension, contraction, bending, or twisting, are achieved with pressure or vacuum supply in combination with common design architectures including: (i) eccentric geometry between fluid chambers and walls (Gorissen et al., 2018); (ii) fabrication with multiple materials of dissimilar properties (Martinez et al., 2013); or (iii) implementation of corrugated internal and/or external geometries (Gorissen et al., 2017). Indeed, many actuator designs have successfully combined these approaches to further enhance their capabilities (Martinez et al., 2013; Matteo et al., 2014; Mosadegh et al., 2014; Ming et al., 2017; Yi et al., 2017).

To exaggerate desirable pressure-strain profiles in single chamber actuators, the elastomeric bodies are often augmented with strain-limiting fiber, mesh, or sheet layers. Fiber reinforcement involves wrapping a pre-molded hollow core with inextensible material (e.g., cotton or Kevlar) before sealing with a second layer of pre-polymer. Inextensible fibers constrain radial expansion of the internal chamber and direct the resultant strain profile of the actuator. Through variation of the winding geometry, precise control over the actuator's behavior is possible (Krishnan et al., 2012), and by combining actuators with differing fiber geometry in series, configurable trajectory matching may be achieved (Bishop-Moser and Kota, 2015; Connolly et al., 2015, 2017; Polygerinos et al., 2015; Kurumaya et al., 2018; Singh and Krishnan, 2020). Though effective, fabricating these actuators is complicated by the need for precision control of fiber path, tension, and adhesion (Agarwal et al., 2016), and resultant devices typically have reduced extensibility and flexibility relative to purely elastomeric structures (Rus and Tolley, 2015). To improve fabrication repeatability and design flexibility, Agarwal et al. (2016) presented a molding approach with pre-formed, integrated reinforcement shells for single-step molding of bending and linear actuators, although this approach does not readily extend to multi-chamber designs.

An alternative fabrication approach, normally allowing greater strain at lower pressures, is to create a network of corrugated chambers within the actuator's body (PnueNets) (Ilievski et al., 2011; Mosadegh et al., 2014; Wang et al., 2018). Conventionally, complex internal and external geometry is first cast in a planar mold followed by bonding of a strain-limiting layer and sealing of the fluid chamber (Schmitt et al., 2018). Large strains may be attained rapidly with this actuator type at relatively low pressures (Mosadegh et al., 2014), and geometric variation again allows modulation of bending behavior (Hu et al., 2018; Wang et al., 2018; Hu and Alici, 2019). These actuators can, however, be susceptible to leakage or failure at the bonded joint (Marchese et al., 2015; Gorissen et al., 2017), and single-step fabrication of chamber geometry is precluded as demolding of ridged cores is often not possible without inducing damage (Galloway et al., 2016). Attempts to mitigate this issue have been presented through the use of soft cores with vacuum extraction (Galloway et al., 2016), rotational casting (Zhao et al., 2015), and sacrificial cores (Marchese et al., 2015; Morley-Drabble and Singh, 2018).

The aforementioned fabrication approaches are typically associated with individual chamber actuators; however, they are also relevant to actuator designs with higher chamber numbers. Unfortunately, this adds significant procedural complexity that is amplified as the device scale is reduced. Generally, multi-chamber actuators employ three chambers distributed with their centers 120° apart (Suzumori et al., 1991a,b; Benjamin et al., 2012; Cianchetti et al., 2013; Martinez et al., 2013; Yahya et al., 2014; Sun et al., 2016; Yan et al., 2016; Drotman et al., 2017; Nguyen et al., 2017; Robertson and Paik, 2017) and may be fabricated in a number of ways; for example: (i) molding with constant axial cross-sectional cores (Suzumori et al., 1997; Martinez et al., 2013; Yahya et al., 2014; Fu et al., 2020); (ii) assembly of pre-formed individual chambers (Cianchetti et al., 2013; Matteo et al., 2014; Ranzani et al., 2015; Nguyen et al., 2017; Garbin et al., 2018, 2019; Peng et al., 2019); and (iii) 3D-printing of integrated designs (Peele et al., 2015; Wallin et al., 2018; Yirmibesoglu et al., 2018; Drotman et al., 2019). Although promising, these methods carry trade-offs between achievable internal chamber geometry, complexity, resilience of assembly, material selection, and practicable actuator scale and feature resolution (Schmitt et al., 2018). Currently, molding-based fabrication offers the most extensive selection of elastomeric materials and is restricted in resolution primarily by the mold manufacturing techniques employed. However, repeatable and simple manufacture of multi-chamber soft actuators, particularly at small scales, remains a challenge and is typically hindered by the necessity for numerous fabrication steps, each reducing the repeatability of the final actuator design.

In this paper we introduce, for the first time, the design concept of “Parallel Helix Actuators” (PHAs). The associated fabrication process we describe allows for the simple production of single elastomer actuators that are capable of three-dimensional mobility at scales <1 cm in diameter. The PHA design overcomes some of the limitations of existing fabrication techniques, particularly those associated with multi-chamber designs. Specific benefits associated with PHAs are (1) integrated multi-chamber designs scalable to small size (<1 cm diameter for three-chamber design); (2) the potential for single-step, single-material molding, reducing reliance on sealing and layer bonding, and avoiding the need for assembly; (3) simple “unscrewing” of mold cores to reduce the risk of damage to the internal features of the actuator during demolding; (4) design flexibility allowing adjustment to PHA geometry and scale for application specific optimization; and (5) 3D motion description through kinematic models based on the constant-curvature assumption. We envisage PHAs having utility in medical robotics for example in minimally invasive surgery (Abidi et al., 2018) or endoscopy (Garbin et al., 2019); or providing a generic actuator platform for soft robotic applications.

Within the following sections we introduce the PHA concept and describe the associated fabrication technique. Through validated adoption of the constant-curvature assumption, we present a kinematic model for PHAs that accounts for the influence of non-linear material properties and inter-chamber variations. Experimental evaluation is reported for varied helix geometries, and 3D workspace characterization with model comparison also presented. Finally, we experimentally investigate the influence of tip loading on achievable workspace and discuss the merits and limitations of PHA in the context of the presented study and alternative approaches.

## Principle of Design

**Figure 1:**
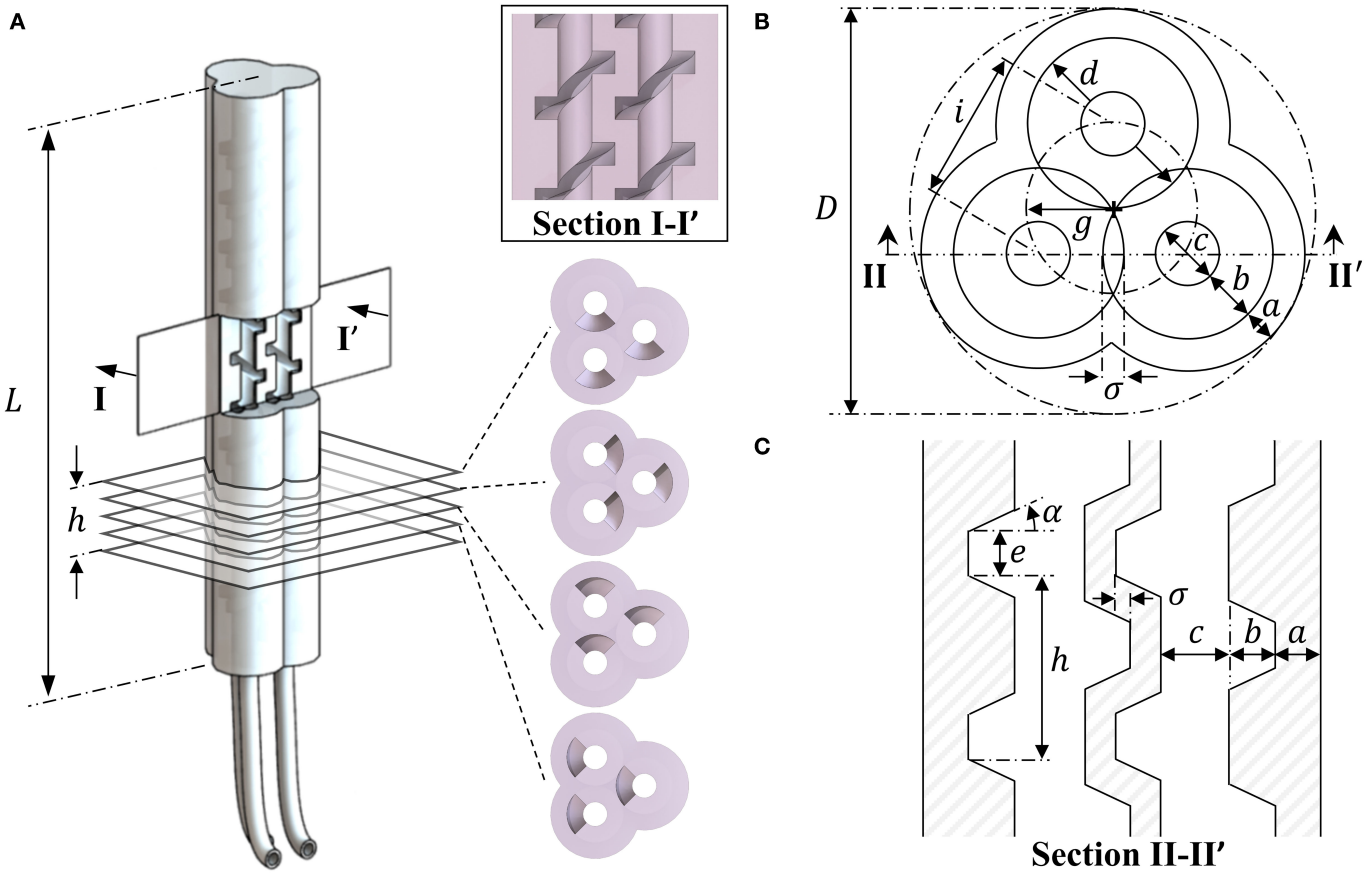
A three-chambered monolithic soft actuator design using parallel cores with helical features, showing **(A)** the completed actuator with chamber and axial cross-sections and dimensional properties for the PHA design associated with **(B)** the axial cross-section and **(C)** the longitudinal features within a sectional view of a plane bisecting the centers of any pair of chambers.

In contrast to many multi-chamber soft actuator designs, PHAs employ helical chamber geometry to form undercuts and corrugations within their internal structure. Figure 1A presents an example PHA design for three chambers aligned in parallel with an even distribution around the actuator's centerline. Helical chambers are produced in an interlocking pattern, requiring them to be of equal axial rotation for all chambers. PHA designs therefore benefit from an anisotropic stiffness distribution produced using only a single elastomeric material, allowing higher strains at low pressure and simplified fabrication. The cross-sectional and longitudinal dimensional parameters of the interlocking helical features are presented in Figures 1B,C respectively, and are described within Table 1.

**Table 1:** Geometric properties of the three-chamber PHA design.

**Variable**	**Definition**	**Influence on overall diameter (D)**	**Values for study**
*a*	Wall thickness	$\frac{\Delta D}{\Delta a}=2$	1 mm
*b*	Thread width	$\frac{\Delta D}{\Delta b}=4.31$	1 mm
*c*	Core shaft diameter	$\frac{\Delta D}{\Delta c}=2.15$	1.5 mm
σ	Thread overlap	$\frac{\Delta D}{\Delta \sigma }=-1.15$	0 mm
*d* = *c* + 2*b*	Core overall diameter	–	3.5 mm
*i* = *d* − σ	Core center spacing	–	3.5 mm
*e*	Thread vertical edge height	–	1 mm
*g*	PHA center to core center distance	–	2.02 mm
*h*	Pitch	–	4 mm
α	Thread horizontal edge angle	–	−25°, 0°, +25°, +50°
	Maximum overall diameter	–	9.54 mm
*H* = (*Nh* + *e* + 2*btan*(α))	Height of the threaded internal structure	–	
*N*	Number of turns	-	10 (no unit)
*L*	Overall length	-	50 mm

Although higher chamber number designs are possible, the minimum required for achieving effective three-dimensional mobility is three. When considering three chamber cores, each of maximum diameter *d*, the overall diameter *D* of the smallest circle that will circumscribe them occurs when they are in a hexagonal packing configuration (i.e., each cotangent with the other two) as given by Kravitz (1967):

(1) $$\begin{array}{l} D=\left(1+\frac{2}{\sqrt{3}}\right)d \end{array}$$

Through inspection of the helical core geometry presented in Figure 1B, Equation (1) may be expanded to give the effective overall diameter for a three-chamber PHA, taking account of the core shaft diameter *c*, thread width *b*, core overlap σ, and external wall thickness *a*, to give:

(2) $$\begin{array}{l} D_{PHA}=\left(\left(1+\frac{2}{\sqrt{3}}\right)\left(c+2b-\sigma \right)\right)+2a+\sigma \end{array}$$

It is evident from evaluation of Equation (2) that the geometric design variables have differing influence on the overall diameter realized. The relative influence on *D* for each of the variables as it is independently increased is presented in Table 1; with unit changes in thread width *b* and thread overlap σ inducing the largest (4.31 mm/mm) and smallest (−1.15 mm/mm) changes in *D*, respectively. In selection of appropriate geometric values, consideration must also be taken to (1) ensure sufficient elastomeric material thickness to avoid excessive ballooning or rupture; (2) achieve suitable structural rigidity of internal mold parts for maintaining accurate alignment during molding and for removal without fracture; and (3) accommodate alignment features beyond the helical geometry of the insert. Design variables must therefore be selected with consideration of the material properties of the actuator and mold components and the mold manufacturing process available.

## Fabrication

**Figure 2:**
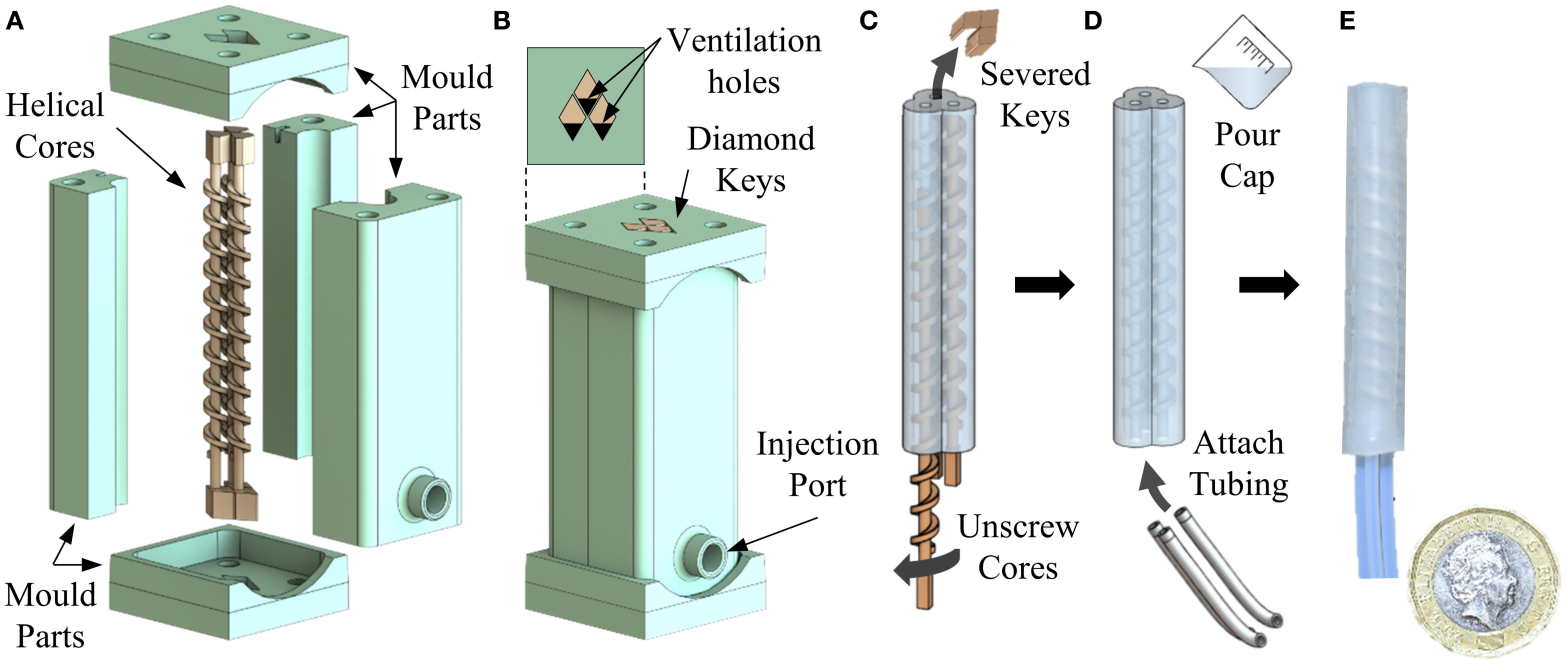
Fabrication steps for producing a three-chamber PHA, showing **(A)** assembly of helical cores and external mold components; including diamond shaped alignment features, **(B)** injection of silicone prepolymer into the assembled mold, **(C)** “unscrewing” of helical cores after curing, **(D)** sealing and attachment of tubing, and **(E)** an example final produced actuator.

Fabrication of PHA designs, in accordance with the dimensions presented in Table 1, was achieved using a molding process as presented in Figure 2. An external five-piece mold design and three helical inserts of desired geometry were printed in resin material (Clear Resin v4; Formlabs, USA) using stereolithography (Form 2 SLA printer; Formlabs, USA). Figure 2A shows the exploded assembly of the mold parts, including the diamond-shaped alignment features added to mold cores and the top and bottom caps allowing precise alignment of internal and external parts. A three-piece split body mold was employed to allow simple assembly and part removal while limiting the influence of seams induced by material flashing at mold joints. A port at the base of the mold was included to allow silicone injection via a standard Luer Lock syringe (Figure 2B).

Silicone prepolymer (Dragon Skin 10; Smooth-On, Inc.) was mixed in equal quantities and degassed under vacuum for 5 min before being injected into the mold using a 1 ml syringe. The silicone was left to cure at room temperature for a minimum of 4 h before demolding from the external mold. Once extracted, the helical cores were removed by first severing the diamond-shaped key from one end and then twisting to *unscrew* from the actuator body from the opposing end (Figure 2C, Supplementary Video 1). Using helical cores reduces the likelihood of inducing damage to the delicate internal features (or the mold cores themselves) during demolding.

**Figure 3:**
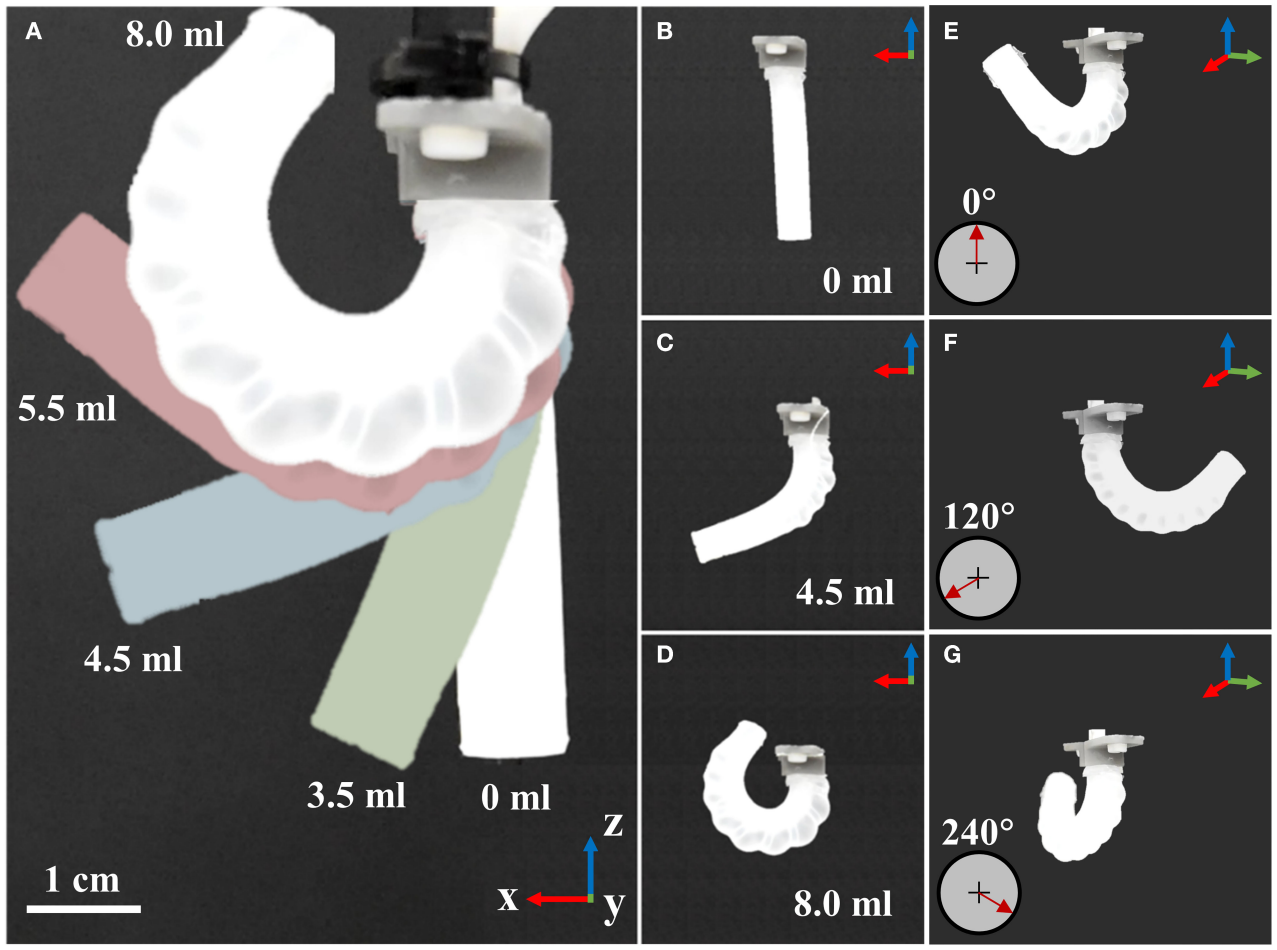
A three-chambered actuator design cast using parallel cores with helical features, showing: **(A–D)** single chamber actuation under varied input volume; and **(E–G)** individual chamber actuation and associated target bending plane direction.

Silicone caps were added to either end of the actuator using the same prepolymer and, once cured, 1 mm diameter holes were punched into the proximal end in line with each chamber. Three tube-to-tube barbed connectors (2808K101, McMaster-Carr, USA) were located into each hole to allow reversible attachment of 1.59 mm (1/16”) internal diameter connecting tubes (Figure 2D) to the actuators. An example resulting PHA actuator for an α = 0° configuration can be seen in Figure 2E. Typical motion behavior achieved under volumetrically controlled pneumatic actuation of individual chambers is also depicted in Figure 3 and in Supplementary Video 1. As seen, the parallel corrugated chambers of PHAs enable simple fabrication of single material actuators with effective in-plane bending performance (>> 180°) and three-dimensional mobility.

### Design Variation

**Figure 4:**
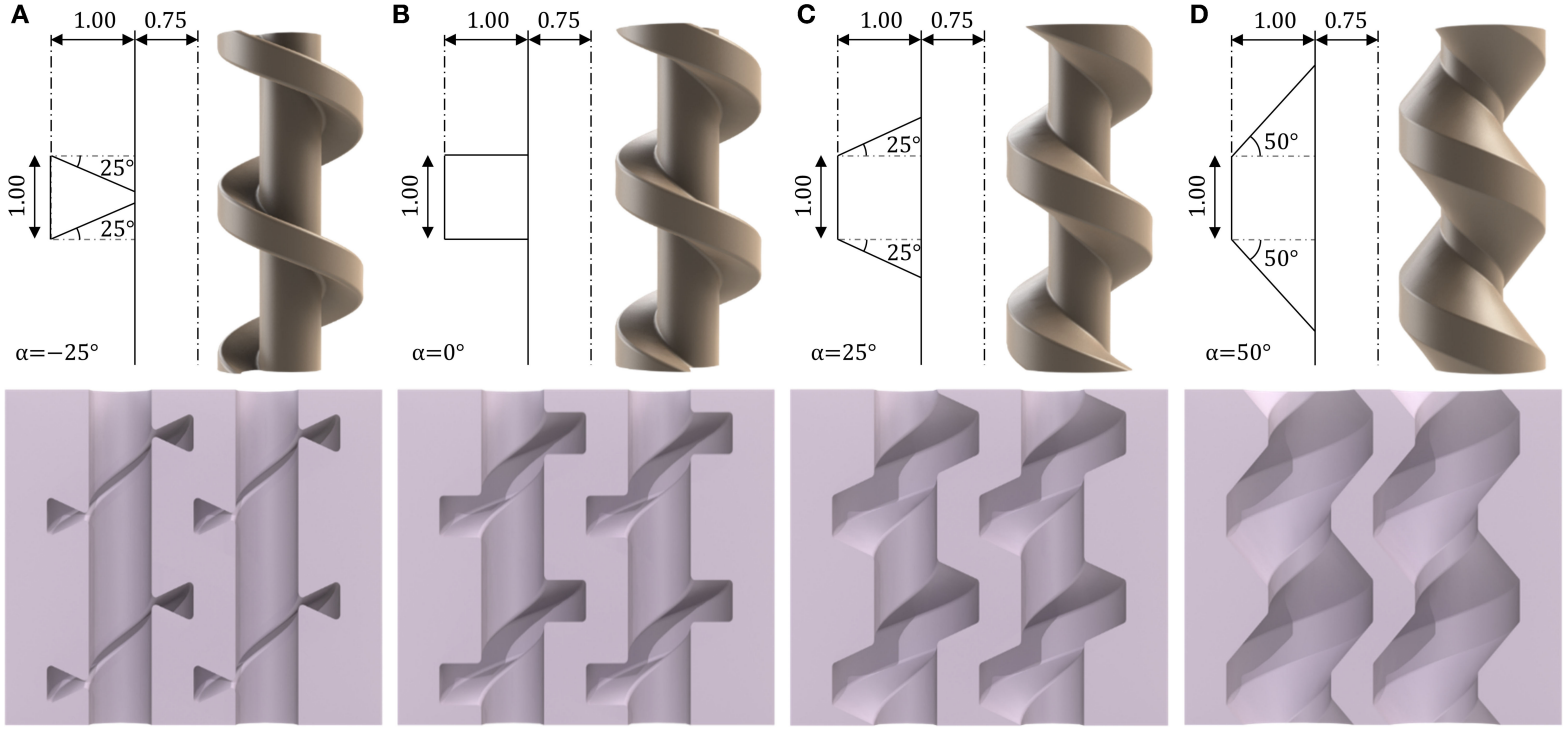
Geometric variation of mold-core thread horizontal edge angle α and corresponding core design and internal cavity of the resultant actuator for α values of **(A)** −25°, **(B)** 0°, **(C)** +25°, **(D)** +50°, units in mm unless otherwise stated.

For the current study, dimensional parameters were selected to produce a < 1 cm diameter design while delivering suitable structural integrity, alignment, and precision of mold cores. Variation of the internal chamber volume for a fixed minimum external wall thickness and overall diameter is possible through adjustment of the horizontal edge angle (α). This parameter was therefore selected to examine its effect on the bending performance of PHA designs. As observed from Table 1, α was chosen to take values from −25 to 50° in 25° increments. Schematic representation of these values and the corresponding thread geometry, mold-core design, and resultant actuator sectional geometry are shown for each in Figure 4.

Cores were constructed from the addition of the spiraling feature at fixed pitch *h* to a central shaft of diameter *c*. To facilitate practicable molding, cores were supplemented with an additional blank length (with no helical feature) of diameter *c* at both ends. The length of this feature was adjusted to achieve a consistent overall length across actuator designs. Diamond-shaped key elements were added to either end of each insert to mate cores with the external mold while ensuring correct orientation, spacing and vertical alignment, as shown in Figure 2A. The resultant PHAs, produced from the parameters in Table 1, deliver overall diameters of 9.54 mm and overall lengths of 50 mm.

## Kinematic Model

Actuation of PHAs is achieved through inflation of the helical chambers, with the center of each being located at a distance *g* from the central axis of the actuator, as shown in Figure 1B. The negative of the core's helical features imprints a spiral of material that runs through each chamber, reinforcing the outer wall and connecting it continuously to the central column of the actuator. It is this feature that, as with fiber-reinforced actuators (Bishop-Moser and Kota, 2015; Connolly et al., 2015, 2017; Polygerinos et al., 2015), acts to constrain expansion radially and thus preferentially promotes elongation of each chamber with applied pressure.

**Figure 5:**
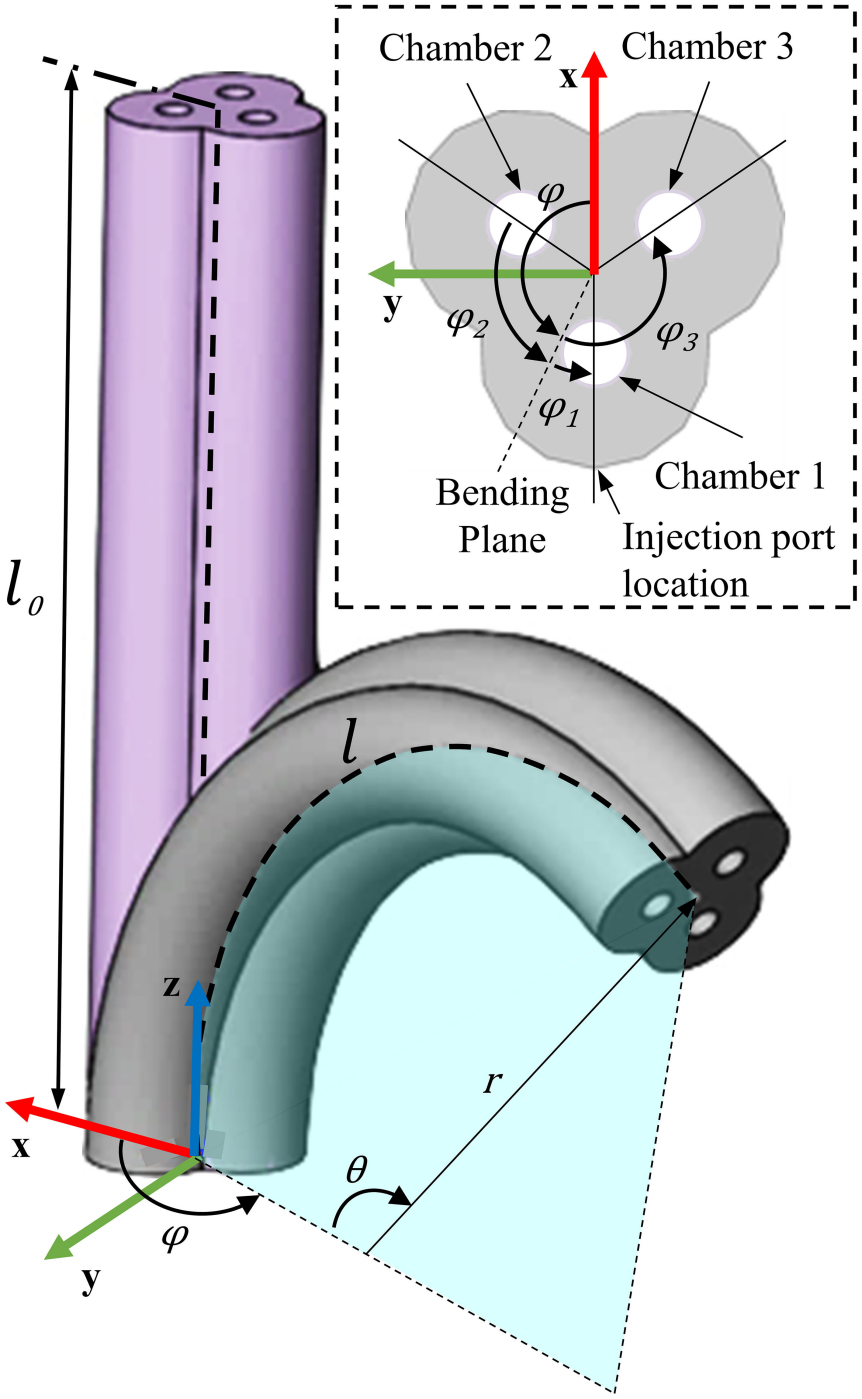
Depiction of bending kinematics for PHAs, showing PHA representation as a circular arc in configuration space (described by bending angle θ, angle of the bending plane φ, and the arc length *l*. Chamber spacing identified within the presented section view, as used in developing the actuator-specific mapping.

Assuming idealized chamber behavior of this nature allows kinematic relationships between the actuator's base frame and tip frame to be derived using the constant curvature approach (Webster and Jones, 2010). For the forward case, the ultimate goal is to have a direct mapping of the inputs to PHA and the position and orientation of the PHA's tip. This typically relies on use of an intermediate configuration space (Simaan et al., 2009; Webster and Jones, 2010), which for the constant curvature assumption, completely describes a circular arc (representing the PHA centerline) using three parameters: bending angle θ, angle of the bending plane φ, and the arc length *l*, as depicted in Figure 5. Two mappings may then be used to describe transition to and from the configuration space, thus completing the forward kinematic chain.

The mapping from configuration space to tip pose (position and orientation in task space) is actuator independent (Webster and Jones, 2010), and may be presented as the homogeneous transformation *T* from the base frame to any frame along the centerline *s*, where *s* ∈ [0, *l*] and θ = κ*s* as:

(3) $$T=\left[\begin{array}{cccc} \text{cos }\varphi \text{ cos }\kappa s & -\text{sin }\varphi & \text{cos }\varphi \;(1-\text{cos }\kappa s) & \frac{\text{cos }\varphi (1-\text{cos }\kappa s}{\kappa }\\ \text{sin }\varphi \text{ cos }\kappa s & \text{cos }\phi & \text{sin }\phi \text{ sin}\kappa s & \frac{\text{sin }\phi (1-\text{cos }\kappa s)}{\kappa }\\ -\text{sin }\kappa s & 0 & \text{cos }\kappa s & \frac{\text{sin }\kappa s}{\kappa }\\ 0 & 0 & 0 & 1 \end{array}\right]$$

where κ is the curvature associated with radius of curvature *r* as κ = 1/*r*. In order to describe the pose of the actuator as a function of the chamber inputs, a device-specific mapping from actuation inputs to configuration space is required. When considering the inputs as the chamber lengths directly, this can be described based on the chamber's geometry relative to the actuator's centerline. Specifically, the centerline length *l* can be related to the individual chamber length as:

(4) $$\begin{array}{l} l=l_{i}+\theta g\text{ cos }\varphi_{i} \end{array}$$

where *l*_*i*_ represents the length of chamber *i* (*i* ∈ [1, 2, 3]), *g* is the distance from the chamber center to the center of the actuator (equal for all chambers), and φ_*i*_ the angle between the bending plane and chamber *i*. For the specific case shown in Figure 5, Chambers 1, 2, and 3 (*C*_1_, *C*_2_, and *C*_3_) are located at angles of 180, 60, and 300 measured counterclockwise from the *x*-axis around the *z*-axis, respectively, resulting in φ_*i*_ values of φ_1_ = 180 − φ, φ_2_ = 60 − φ, and φ_3_ = 300 − φ. Consequently, $\overset{3}{\underset{i=1}{\sum }}\text{cos }\varphi_{i}=0$ which when combined with Equation (4) leads to:

(5) $$\begin{array}{l} l=\frac{l_{1}+l_{2}+l_{3}}{3} \end{array}$$

As detailed by Webster and Jones (2010), expressions may also be developed for determining the bending plane φ and the curvature κ, as shown in Equation (6) and (7), respectively.

(6) $$\varphi =\;{\text{tan}}^{-1}\left(\frac{\sqrt{3}(l_{2}+l_{3}-2l_{1})}{3(l_{2}-l_{3})}\right)$$

(7) $$\begin{array}{l} \kappa =\frac{2\sqrt{l_{1}^{2}+l_{2}^{2}+l_{3}^{2}-l_{1}l_{2}-l_{1}l_{3}-l_{2}l_{3}}}{g\left(l_{1}+l_{2}+l_{3}\right)} \end{array}$$

For the presented device-specific mapping, this only extends to the consideration of chamber lengths, which may be used only directly to describe the actuator kinematics when the inputs to the chambers are considered proportional to their length (Suzumori et al., 1991b; Abidi et al., 2018). PHA elongation is achieved through the development of appreciable strain within the thin walled regions of the external face (Figure 3A) and, as such, the elongation is subject to the non-linear stress-strain relationship associated with the elastomeric material (Moseley et al., 2016). Additionally, the actuation presented is achieved through pneumatic volumetric control that introduces the influence of air compressibility into the system. In combination, these aspects render a directly proportional input-to-elongation assumption invalid for the PHA. Alternatively, we propose that the chamber length be described as a function of the chamber *i* input volumes *v*_*i*_, as:

$$\begin{array}{l} l_{i}=f\left(v_{i}\right). \end{array}$$

Rearranging Equation (4), assuming an invariant centerline length *l*, and considering uniaxial bending cases only, i.e., *cosϕ*_*i*_ = −1, gives:

(8) $$\begin{array}{l} l_{i}\left(vi\right)=l+\theta \left(v_{i}\right)g^{*} \end{array}$$

As *g* represents the distance from the centerline of the PHA to the chamber center under the assumption of no radial deformation, it represents a potential source of error within the kinematic model. To account for this, a new variable, g^*^, is introduced in Equation (8), which represents an approximation of the mean distance from PHA centerline to chamber centers over the full actuation range.

With the chamber lengths *l*_*i*_ described as a function of the input volume *v*_*i*_, Equations (3–6) may be used to describe the forward kinematics for the PHA. However, due to the helical shape of the chamber walls, an additional twist factor must be accounted for in the kinematic model. This may be simply applied as an additional rotation around the base *z*-axis by an angle φ^*^, as

(9) $$\begin{array}{l} T_{PHA}=\left[\begin{array}{cccc} \text{cos }\varphi^{*} & -\sin\varphi^{*} & 0 & 0\\ \sin\varphi^{*} & \text{cos }\varphi^{*} & 0 & 0\\ 0 & 0 & 1 & 0\\ 0 & 0 & 0 & 1 \end{array}\right]T \end{array}$$

where *T*_*PHA*_ represents the transformation specific to the PHA design. As the presented kinematic model is based on the constant-curvature assumption, we first experimentally investigate its suitability when applied to PHAs. Subsequently, we present strategies for determination of a bending-volume function and rotation offset φ^*^.

## Experimental Evaluation

Once fabricated, the single chamber bending performance of the four PHA design variations presented in Figure 4 was evaluated for comparison under volumetric control. Based on the superior bending performance demonstrated for the PHA with α = 0, further investigation of this design was performed to determine suitability of the constant curvature model and to calibrate the chamber length functions for application in a full 3D kinematic model. Furthermore, open-loop bending performance was assessed across a range of speed (volume rates) and control scenarios. Coupled multi-chamber control was subsequently performed to understand the achievable 3D workspace and for comparison with the kinematic model. Finally, the carrying capability of the design was subsequently assessed through investigation of the impact of tip loading on the achievable workspace.

### Uniaxial Characterization

**Figure 6:**
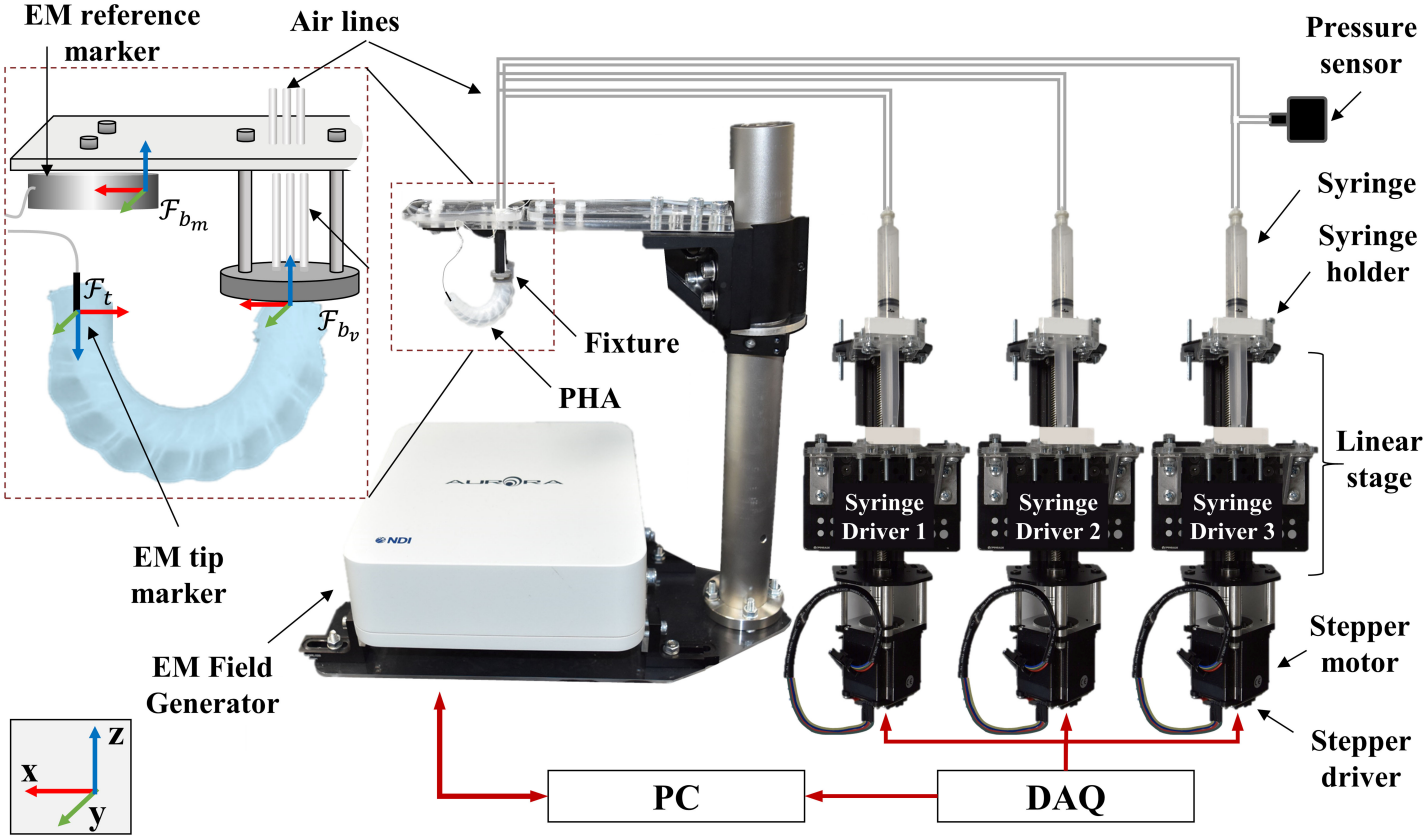
Experimental setup used for bending and workspace performance assessment, showing frames $F_{b_{m}}$, $F_{b_{v}}$, $F_{t}$ representing measured base, virtual base, and tip frames, respectively; scale adjusted for clarity.

Primary evaluation of actuation performance was conducted by supplying air to each of the three chambers independently, while monitoring the position and orientation of the actuator tip. Bending tests were performed independently under volumetric control, using an experimental setup as illustrated in Figure 6. Each candidate PHA was mounted securely using a custom 3D-printed fixture (Gray Pro; Formlabs, USA) that conforms to the external geometry of the actuator base. A small hole (Ø1 mm) was punched centrally into the cap of the actuator and an electromagnetic sensor (Aurora Micro 6DOF Sensor Tool, NDI, Canada) was inserted into the cavity. Motions of the tip were subsequently recorded relative to a base frame sensor (Aurora 6DOF Reference disc, NDI, Canada) using an electromagnetic tracking system (Aurora Planar 20-20 V2, NDI, Canada) sampling at 40 Hz.

To control the volume of air delivered to each chamber, custom syringe driver units were developed based on a lead screw linear actuator (V-Slot® NEMA 23 Linear Actuator, Openbuilds, USA) and laser-cut acrylic components. Stepper motors were controlled via driver boards (uStepper S-lite, uStepper Aps, Denmark), embedded software, and digital signal interface (NI 9401, National Instruments, USA) controlled via software (LabVIEW, National Instruments, USA). Acrylic components (5 mm thick RS PRO, RS Components, UK) were attached to the actuation frame and tray to accommodate control of a standard 10 ml syringe. The assembled syringe driver units are presented in Figure 6.

Each syringe displaces 0.153 ml.mm^−1^ and when coupled to the 400 step.mm^−1^ linear actuator produces a theoretical volumetric resolution of 0.38 μl. Initial bending tests were performed through linear injection of air into the chamber under test at a rate of 1.6 ml.s^−1^ up to a total volume of 8 ml, predetermined to achieve bending angles of >180° for the specific actuator-chamber combinations. The tip position and orientation of each actuator-chamber combination was recorded for three repeats along with the chamber pressure; measured using a pressure transducer (40PC100G2A, Honeywell International Inc., USA) through a data acquisition board (USB-6211, National Instruments, USA).

The measured base frame $F_{b_{m}}$ was translated virtually to account for the geometric offset at its mounted location relative to the actuator holder, resulting in a virtual reference frame $F_{b_{v}}$ at the base of the actuator. The tip frame $F_{t}$ was corrected for misalignment of the inserted electromagnetic sensor at the tip and oriented to the base frame using:

$$\begin{array}{l} Q_{tip}\left(t\right)=Q_{tip}^{*}\left(t\right)*\left({Q_{tip}^{*}\left(0\right)}^{-1}*Q_{base}\left(0\right)\right) \end{array}$$

where *Q*_*tip*_(*t*) and $Q_{tip}^{*}\left(t\right)$ represent the aligned and measured tip frame quaternions at time *t*, respectively; ${Q_{tip}^{*}}^{-1}\left(0\right)$ and *Q*_*base*_(0) represent the inverse of the measured tip and base quaternions at ambient pressure (i.e., *t* = 0), respectively; and * represents the quaternion product. The bending angle of the tip θ was determined through a global rotation of the *z* vector of the tip $\left(F_{tz}\left(i\right)=Q_{tip}\left(i\right)F_{tz}\left(0\right){Q_{tip}\left(i\right)}^{-1}\right)$ to align closely with the positive *x*-axis of the base frame $F_{b_{v}x}$, followed by taking its projection in the *x-z* plane and determining the angle relative to the global *z*-axis.

**Figure 7:**
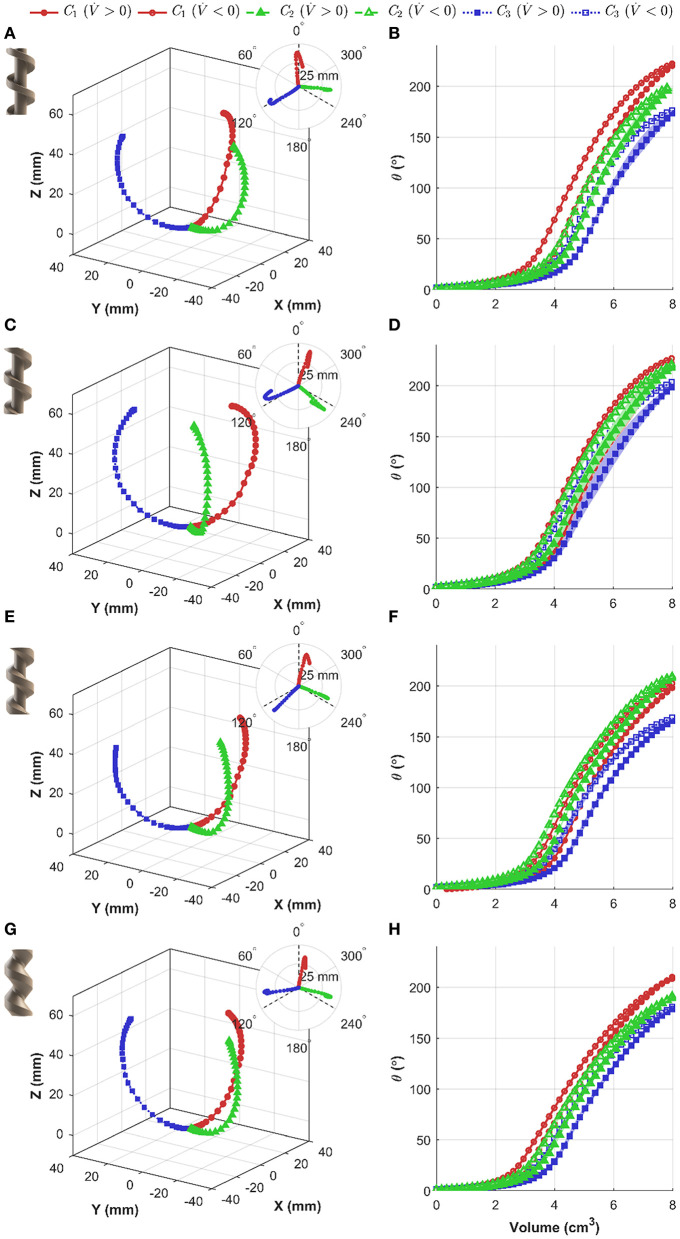
Independent chamber actuation testing under volumetric control for varied actuator designs, showing (left) actuator tip position (including *x-y* plane view insert), and (right) bending angle θ as a function of applied volume (shaded regions represent the bounding range from three repetitions); design values for α of: **(A,B)** −25°, **(C,D)** 0°, **(E,F)** +25°, and **(G,H)** +50°.

Figures 7A,C,E,G show the Cartesian coordinates of the tip frame for each PHA under test, also indicating the achieved bending planes and bending angles corresponding to each chamber. Figures 7B,D,F,H show bending angle vs. increasing and decreasing volume for three repeats of each chamber. It is evident that for small added volumes, the bending angle increases moderately and in an approximately linear fashion. Above bending angles of ~15°, a rapid increase in θ occurs with subsequent addition of air. The identified transition volume of air and corresponding pressure both increase as the value of α reduces.

To allow more direct comparison between the internal geometries tested, the maximum bending angle and peak pressures have been summarized in Table 2. It is evident that there is a consistent trend in maximum bending angle and peak pressure as a function of chamber number. For increasing chamber number, the maximum angle decreases for all PHAs; however, for the α = 0 design variability is less prominent, and the maximum bending angle is highest for each chamber relative to other designs. In general, the reducing internal volume associated with a reduction in α results in an increase in peak chamber pressures.

**Table 2:** Comparison of mean ± SD (*n* = 3) values for varied PHA internal geometry.

**Thread horizontal edge angle, α (*^**°**^)***	**Chamber no**.	**Max θ (^**°**^)**	**Peak pressure *(kPa)***
−25	1	222 ± 0.6	75.6 ± 1.4
	2	202 ± 1.8	82.0 ± 3.2
	3	176 ± 0.9	87.1 ± 2.4
0	1	227 ± 0.9	62.5 ± 2.5
	2	222 ± 1.7	62.6 ± 2.2
	3	204 ± 0.7	64.3 ± 2.3
25	1	209 ± 1.3	65.4 ± 2.2
	2	202 ± 0.3	69.9 ± 1.9
	3	168 ± 0.8	73.7 ± 1.8
50	1	210 ± 0.5	47.6 ± 0.3
	2	191 ± 0.1	51.3 ± 0.3
	3	181 ± 0.3	53.5 ± 0.2

### Chamber Modeling

Utilizing the tip frame transformation *T*, Equation (3), a predicted tip position p^*^(θ, *l*, φ) may be determined. To understand the efficacy of the constant-curvature model for describing the behavior of the PHA's chambers, a least-squares optimization approach was taken using the measured tip position data. The input values of bending angle θ and Cartesian position **p** were used along with the Levenburg–Marquardt method (Moré, 1978) to minimize the sum of the squares of the deviations *S*(**β**), where:

(10) $$S\left(\beta \right)=argmin_{\beta }\sum_{j=1}^{m}{\left[{\text{p}}_{j}-{\text{p}}_{j}^{*}(\theta_{j},\beta )\;\right]}^{2}.$$

**Figure 8:**
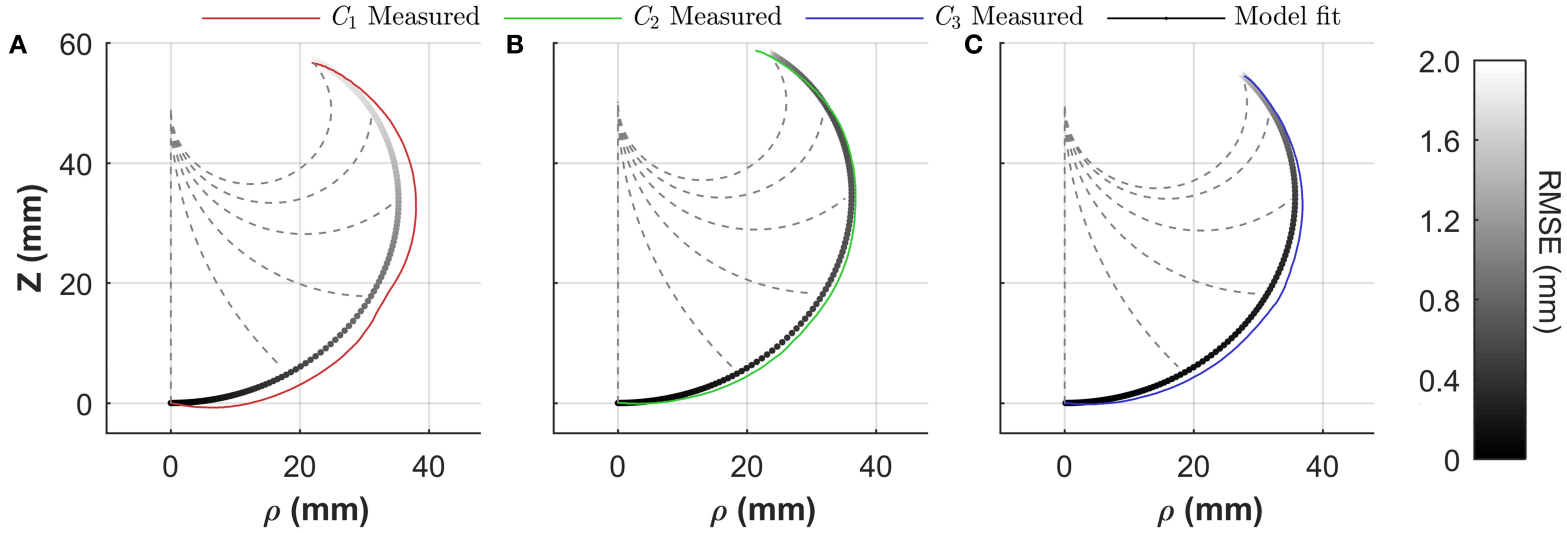
Constant curvature model fitting performance for an α = 0 actuator for **(A)** Chamber 1 (*C*_1_), **(B)** Chamber 2 (*C*_2_), and **(C)** Chamber 3 (*C*_3_); grayscale model fit color maps represent the RSME for the fit as a function of bending angle θ; dashed lines show modeled shape for 0, 45, 90, 135, 180°, and maximum bending angle.

In this case **p**_*j*_ and ${\text{p}}_{j}^{*}$ represent the measured and predicted tip positions at measurement point *j*, respectively, and **β** the vector of the optimization variables arc length *l* and bending plane φ. Model fitting was repeated with an increasing range of θ values, up to the maximum recorded bending angle, i.e., *m* ∈ [1, *m*(θ_max_)]. Figure 8 shows the achieved constant curvature fit in cylindrical coordinates as determined from the full range of bending angles (i.e., *m* = *m*(θ_max_). It is evident that the constant curvature approximation at maximum bending yields strong conformation with measured data. To assess performance as a function of bending, the Root Mean Square (RMS) error between the measured and modeled tip position for fitting results, determined as a function of the bending angle, is overlaid as a color map onto the model fit for each chamber in Figure 8, showing a maximum deviation of 2 mm within Chamber 1 at maximum bending angle.

Fitting in accordance with Equation (10) also allows optimal identification of the plane of bending φ and effective arc length *l*. Table 3 presents the average (mean ± SD) values from the optimization for both parameters and for each chamber, indicating small variability in arc length with volume in all cases, thus justifying the assumption of invariant length required for formulation of Equation (8). In addition, offsets are evident between chamber positions and their realized bending planes, although variability is again low. These values may be unitized in generating suitable approximation of φ^*^ to be applied in Equation (9). For the presented 3D model in the following section, φ^*^ was taken as the mean bending plane offset across all chambers (φ^*^= 18°).

**Table 3:** Model fit parameters for bending angle-volume relation for three PHA chambers.

**Chamber no**.	**l (mm) Mean ± SD**	**φ (^**°**^) Mean ± SD**	***A*_1_**	***B*_1_**	***E*_1_**	***A*_2_**	***B*_2_**	***E*_2_**	***R*_2_**
1	49.9 ± 2.8	−14.5 ± 4.5	4.10	8.82	3.45	0.415	5.45	1.08	>0.99
2	50.0 ± 0.9	231.2 ± 3.7	4.13	9.71	3.86	0.424	6.03	1.50	>0.99
3	48.3 ± 2.4	116.5 ± 4.5	4.38	7.69	2.81	−0.960	7.01	1.28	>0.99

To determine suitable relation between bending angle and volume for Equation (8), data from Figure 7D under positive volume rate were fit with a two-term Gaussian Model (*K* = 2), in the form:

(11) $$\begin{array}{l} \theta =\overset{K}{\underset{k=1}{\sum }}A_{k}e^{-{\left(\frac{\text{v}-B_{k}}{E_{k}}\right)}^{2}} \end{array}$$

where *A*_*k*_, *B*_*k*_, and *E*_*k*_ represent function coefficients for the Gaussian term *k*. Table 3 presents the determined model coefficients for the three chambers including the model fitting accuracy, *R*-squared (*R*^2^). The applied model of Equation (11), although generic, captures the volumetric dependence of the bending angle to a high degree of accuracy for all chambers.

### Open-Loop Performance

**Figure 9:**
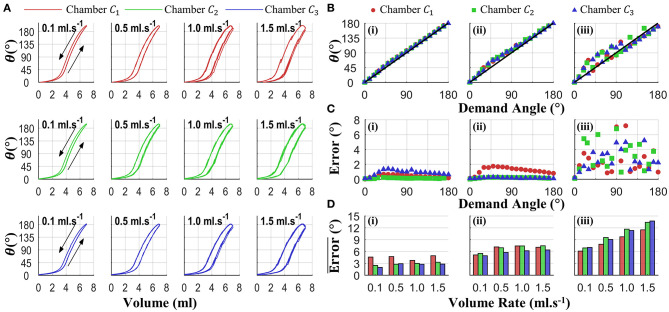
Independent chamber actuation testing for varied volume rates (0.1, 0.5, 1, 1.5 ml.s^−1^); showing **(A)** bending-volume response for each chamber-volume-rate combination (arrows denote volume-rate direction), **(B)** mean open loop performance (five repeats) for tests at 1.5 mls^−1^ under control conditions of (i) sequential quasi-static, (ii) random quasi-static, and (iii) random with minimal (100 ms) delay (diagonal black line representing ideal linear open-loop performance), **(C)** the associate variability (standard deviation), and **(D)** summary of mean errors across all angles for each volume-rate and control condition (5 repeats).

To further understand PHA performance under representative conditions, additional individual chamber testing was conducted. Specifically, the influence of volume-rate on bending performance was first assessed through inflation of each chamber up to and down from 7 ml for 5 repeats at volume-rates of 0.1, 0.5, 1.0, and 1.5 ml.s^−1^. The resultant 5 bending angle vs. volume loops for each condition are shown in Figure 9A. It is evident that a high level of repeatability is present for all test conditions, and an increased volume rate leads to greater hysteresis in the chamber response.

To determine the influence of this behavior on PHA bending under open-loop conditions, a set of demand volumes were selected to correspond to bending angles from 0 to 180° in 10° increments angles; specifically associated with the 0.1 ml.s^−1^ test case under increasing volume. These demand angles were then supplied to the drive system using three different open-loop conditions: (i) sequential quasi-static, constituting a sequentially increasing demand angle with a 5-s hold at each prior to recording the bending angle; (ii) random quasi-static, same conditions as in (i) with demand angles in a random input order; and (iii) random dynamic, same demand sequence as in (ii) with a minimal delay between angles (100 ms). For each chamber, volume rate, and control mode configuration, 5 repeats were performed. Figure 9B shows an example measured angle vs. demand angle for the fastest volume rate (1.5 ml.s^−1^, representing the worst case volume rate condition) under the three control conditions, the diagonal line illustrating the ideal open loop response. The corresponding variability (standard deviation) for this test is presented in Figure 9C. In combination, it is apparent that quasi-static conditions—i.e., (i) and (ii)—offer improved performance with respect to minimal delay between demand angles, i.e., (iii). Figure 9D summarizes the mean errors across all angles under each chamber and test condition, further illustrating the improvement with quasi-static conditions and, to a lesser extent, slower volume rates.

### Multiaxial Characterization

**Figure 10:**
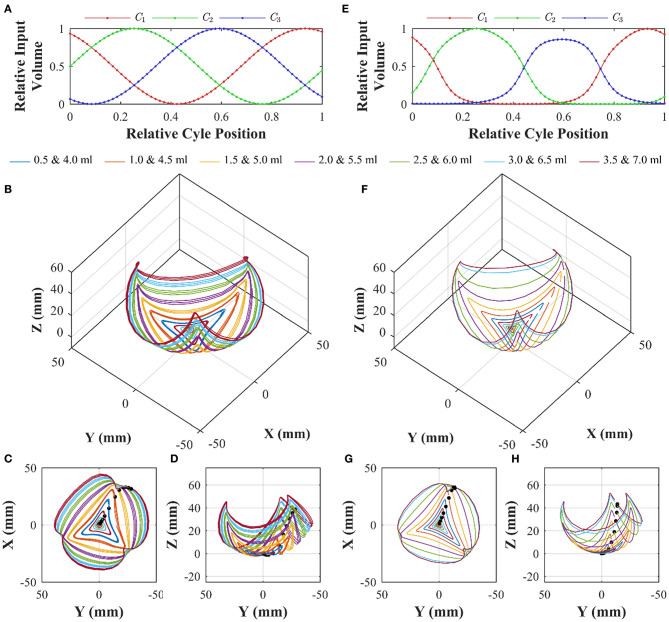
Workspace characterization of an α = 0 PHA under sinusoidal volumetric input of the form **(A)**; showing 3D tip position under three cycles for peak volumes ranging from 0.5 to 7.0 ml **(B)**, with planar views in **(C,D)**; and equivalent model predicted chamber lengths **(E)** and corresponding modeled tip positions under the same actuation inputs in 3D **(F)**, and planar views **(G,H)**. Black dots indicate starting positions for successive maximum volume tests; with cycles progressing in a clock-wise direction with respect to the *x-y* plot.

Multi-axial testing was performed to understand the 3D workspace of the PHA under combined chamber actuation. Based on a linear three-chambered system (i.e., an actuator where chamber lengths are direct inputs from the external actuation system), control inputs of phase-separated sine waves with a phase separation of 120° should produce a rotation around the base frame *z*-axis, i.e., cyclic variation of φ and a constant bending angle dependent on the amplitude of the sine wave. This input signal, as shown in Figure 10A, was therefore set as a drive input for the PHA. An offset and amplitude were set for independent tests at maximum chamber volumes ranigng from 0.5 to 7.0 ml in 0.5 ml increments. The experimental setup shown in Figure 6 was used for testing, with each cycle being discretized into 60 steps, and a total of 3 cycles were performed at each volume (180 data points total). Each chamber-volume combination was employed as a discrete control input to the syringe drivers, and a settling time of 2 s was allowed prior to recording the associated tip pose, representing a quasi-static situation comparable to control case (ii) detailed above.

The resulting tip positions for each volumetric test are presented in Figures 10B–D. The traces indicate that increasing the maximum chamber volume results in a greater mean bending angle. However, the chamber-dependent length-volume properties are also evident, resulting in asymmetrical motion. Furthermore, with continued actuation a slight drift inward (toward the non-actuated tip position) occurs (a maximum tip deviation of 3.3 mm across all cases was determined), resulting from air losses within the system.

Chamber modeling, as presented in the previous section, was employed to convert actuator space sinusoidal drive signals into the equivalent chamber lengths using Equation (8) in conjunction with model coefficients determined in Table 3. The resulting, normalized chamber length variations for the maximum volume test at 7.0 ml are shown in Figure 10E. It is apparent that the non-linear input volume to chamber length relation and chamber variability impart a large influence on the effective chamber lengths seen during equal amplitude (of actuator drive) actuation. Through assessment of the absolute error between measured (for cycle 1 only) and modeled data points, an optimal value of g^*^ was determined to be 3.5. As detailed previously, this is considered as a correction to relax the assumption of an invariant distance between the effective chamber center and the actuator's central axis; necessary to account for small levels of radial expansion. With the selected model parameters, the 3D workspace prediction for the same experimental test condition is as shown in Figures 10F–H.

The model shows a high level of agreement with the measured data, capturing the workspace and chamber biases. To evaluate the model performance quantitatively, the RMS tip error was evaluated for each peak volume test along with its variability (Table 4). Across all volumes tested, a mean RMS tip error of 4.1 mm was determined. It is evident that with increased peak volumes the variability increases as a result of the larger range of effective bending angles during each cycle.

**Table 4:** Root Mean Square tip error (Mean ± SD) for 3D model vs measured data recorded across volumetric range from 0.5 to 7.0 ml.

**Peak supplied volume (ml)**													
**0.5**	**1.0**	**1.5**	**2.0**	**2.5**	**3.0**	**3.5**	**4.0**	**4.5**	**5.0**	**5.5**	**6.0**	**6.5**	**7.0**
4.57 ± 0.01	4.54 ± 0.04	4.41 ± 0.10	4.21 ± 0.15	4.00 ± 0.24	3.67 ± 0.38	3.26 ± 0.57	2.91 ± 0.84	3.09 ± 1.45	4.00 ± 2.20	4.60 ± 2.50	4.35 ± 2.55	4.51 ± 2.84	5.26 ± 3.68

### Influence of Tip Loading

**Figure 11:**
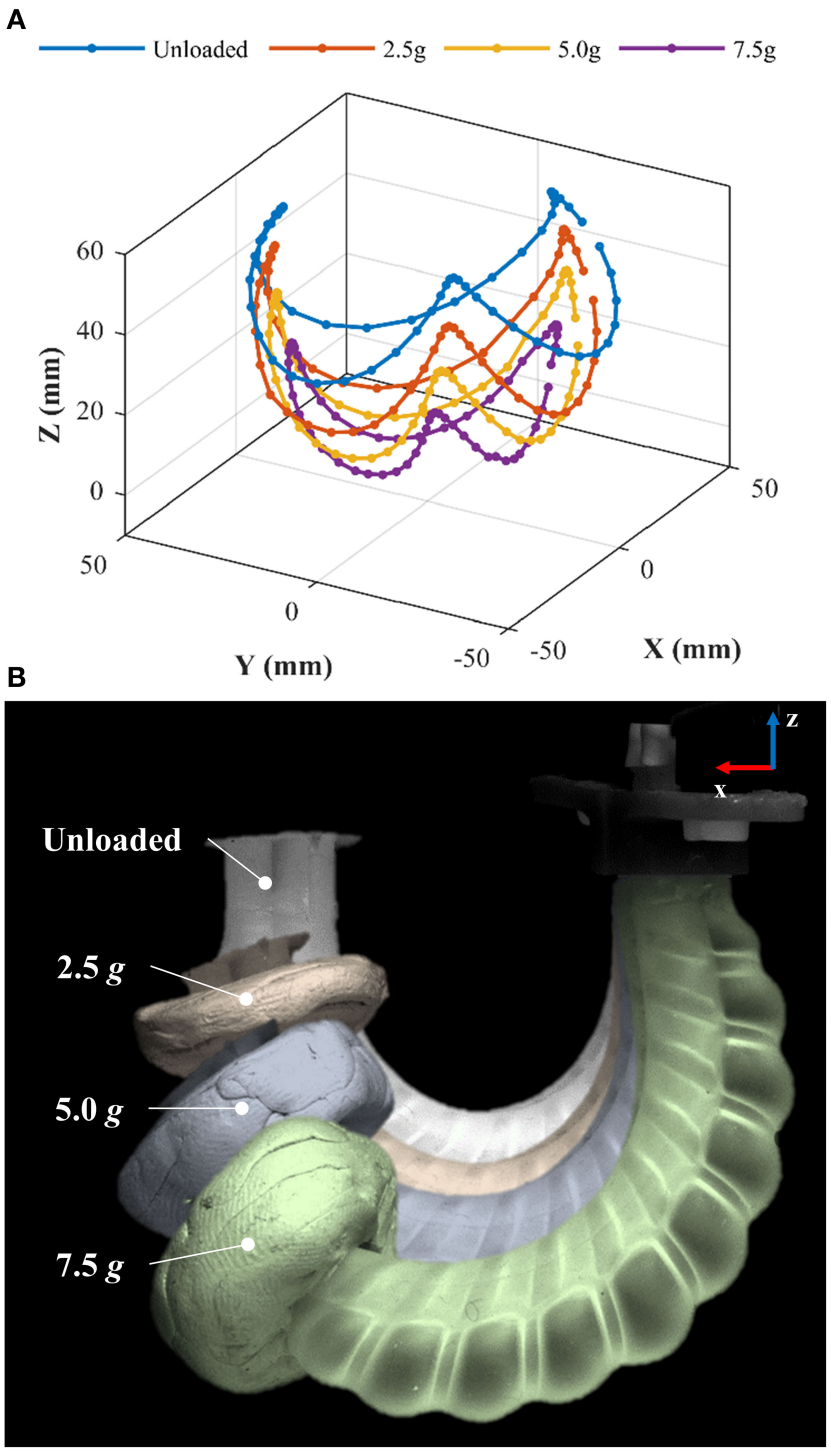
Workspace characterization of an α = 0 PHA under sinusoidal volumetric input of maximum 7 ml and varied tip loads showing **(A)** the 3D tip motion data and **(B)** overlays of PHA with varied tip loads when bending in the *x-z* plane.

Loading of the PHA design may be required for carrying addition functional components (e.g., an endoscopic camera or surgical tool); serially stacking PHAs for increased DoFs, or manipulating payloads. To evaluate the change in performance of the PHA when loaded, workspace characterization tests were conducted with the inclusion of increasing tip loads. Masses of 2.5, 5, and 7.5 g were formed from putty adhesive (Blu-Tack, Bostik, USA) into even cylindrical geometries and attached to the proximal end of the PHA in the non-actuating region; masses selected represent ~1-, 2-, and 3-times bodyweight, respectively (actual bodyweight of tested PHA was 2.47 g). Characterization was subsequently performed using the experimental setup presented in Figure 6, with input volumes of 7 ml maximum in the relative sequence presented in Figure 10A. The determined workspaces from the loads tested are shown in Figure 11A and within Supplementary Video 2. It is evident that the increased load reduces the bending achieved for the same volume, as can be seen on the overlay comparison in Figure 11B. However, the achievable workspace follows a similar form as in the unloaded case and maintains adequate 3D coverage, even at the largest load tested, suggesting suitability of PHAs for loaded applications up to 3-times bodyweight.

## Discussion

The bending performance for monolithic designs, (e.g., Suzumori et al., 1997; Cianchetti et al., 2013; Yahya et al., 2014), is typically limited, suffering from ballooning effects resulting in large radial expansion and an associated reduction in achievable bending angles. To overcome these limitations, designs often incorporate constraining mechanisms through the joining of multiple materials; either circumferentially (Suzumori et al., 1991a; Cianchetti et al., 2013; Yahya et al., 2014; Yan et al., 2016; Abidi et al., 2018) or axially (Martinez et al., 2013). However, to realize these designs a discontinuity in material properties is necessary, the integrity of which depends on the bonding compatibility and strength between the dissimilar materials, and the inclusion of which can drastically increase fabrication complexity. In this article we avoid discontinuities and present a concept, fabrication method, and kinematic modeling approach for multi-chamber monolithic soft actuators based on parallel alignment of helical cores. The use of helical cores has the benefits of (1) producing wall profiles that restrict radial expansion and thus promote chamber elongation and actuator bending and (2) allowing compact layout and removal without damage. The presented PHAs target a sub 1 cm diameter with three parallel chambers, and demonstrate the possibility to achieve large bending angles (> 180°) that closely conform to a constant curvature approximation, and the possibility to support loads up to 3-times bodyweight. The level of bending and the 3D workspace achieved are considerable, and is unmatched for comparable single material designs.

The high number of design variables (Table 1) coupled with the range of possible fabrication materials presents significant opportunity for customization. We present design augmentation in terms of helix fin angle α which introduces varied chamber volumes for consistent external dimensions, resulting in varied volume-, and pressure-bending angle responses. A distinction associated with the use of a helical core profile (vs. a constant cross-section core) is the introduction of torsional effects. For the presented PHA designs, this presents a rotation of bending planes with respect to chamber locations, and a chamber specific volume-bending response. For the former, this effect is limited by the solid central axis of the PHA produced using separated helical cores, which acts to increase the torsional stiffness of the actuator. Adequate mitigation in this case has been proposed through a simple rotational offset, although this may be further extended to be volume- and chamber-specific as required. With more drastic modification of PHA design parameters, for example, reducing material stiffness; increasing helix pitch, fin width, or length; or more closely aligning cores may exaggerate torsional effects. This property can be used to good effect, for example, to improve grasping performance (Hu et al., 2018; Hu and Alici, 2019); however, it would require adaptation of the presented modeling approach to accommodate this behavior. The second effect of using a helical core design is a consistent trend between chamber number and bending angle at maximum volume (Table 2). It is proposed that this is a result of the use of identical threaded cores aligned in equal axial rotation. This condition alters the effective area of thin walled regions along the external face of each chamber and therefore its elongation as a function of input volume. However, using the chamber modeling approach presented, volume-length relationships were determined for each chamber thereby accounting for this factor along with the influence of non-linear elastic properties and air compressibility.

Testing presented in Figure 9 demonstrates the influence of volume-rate on bending performance, as well as its effect on open-loop angle control for varied actuation strategies. This highlights a limitation in using pneumatic volumetric control as an appreciable volume-rate dependence. However, for quasi-static conditions close adherence to an ideal linear open-loop system is evident, with mean angle errors remaining below 8° with high repeatability (SD > 2°), making this a suitable approach for certain applications under open-loop control, while highlighting the potential for improvement with a closed-loop pressure control implementation.

Applying individual chamber models to the 3D case and utilizing the constant curvature assumption, generates a quasi-static approximation of the PHA's 3D motion with a maximum RMS tip error of 5.26 mm. This maximum error approximates the PHA radius, and as such may support open-loop control application where this level of accuracy is acceptable and disturbance is minimal. Importantly, repeatability under cycled actuation remains higher (maximum of 3.3 mm across all cases), indicating that the dynamic effects, which are not considered in the model, do not greatly influence undisturbed quasi-static conditions, something notable from individual chamber testing in Figure 9. This chamber characterization and modeling approach may also be applied to alternative designs where consideration of the non-linear chamber length response during pressure/volume actuation is appreciable.

For the case presented, pneumatic volume control was employed to deliver stable actuator positions. Direct pressure control is problematic in this case due to the highly non-linear pressure-volume response introduced by the high strain levels of the body elastomer. This means that small pressure changes can result in large volume changes (and resultant bending), leading to risk of over inflation (ballooning), and even rupture. Preforming volume control with air introduces undesirable compressibility effects which require mitigation (e.g., through the use of the presented model characterization approach). For more practical implementation of PHAs direct pressure control is desirable; however, it necessitates the implementation of safety considerations and should be ideally employed under closed-loop control. This may be achieved through integration of onboard sensing and may allow, for example, higher accuracy and precision in tip positioning, contact detection, and autonomous motion routines.

The load carrying capacity of the presented PHA of three times bodyweight (7.5 g) (Figure 11 maybe useful for certain applications at this design scale (e.g., <1 cm diameter). For example, carrying a lightweight CMOS camera for endoscopic tip articulation. Furthermore, using an assembly of PHAs to produce soft robots for grasping or locomotion applications may enhance carrying capacity. However, beyond this, the use of stiffer materials, high-output-force, larger designs, and the inclusion of additional strain limiting elements may be explored to improve PHA force output. The PHA version presented also requires a secondary sealing processes at its non-functional ends due to the entry and exit of the threaded cores. This is a consequence of the need for precision alignment and the limited tolerance of the printed parts used. If parallel alignment of cores may be maintained from a single-ended constraint (e.g., for larger scale and/or shorter designs or with improved tolerances of mold parts through better printing or machining), then the need for secondary sealing can be avoided, allowing a truly single-step mass-fabrication process to be realized.

## Conclusion

The PHA design aims to engender simple, repeatable fabrication for small scale multi-chambered soft actuators. This generic type of actuation unit has potential use across many soft-robotic application areas, e.g., endoscopic devices, soft locomoting robots, and compact gripper designs. The principle of the design is compatible with the wide range of elastomeric and mold materials. Further research may focus on the scalability of PHAs, taking them in parallel and serially stacked configurations for increased degrees of freedom, as well as at larger and smaller diameters. In addition, the potential for employing more chambers per actuation unit as well as designing tapered and/or non-parallel implementations will be investigated to deliver altered bending and torsional kinematics and improved carrying capacity. Finally, partial or complete automation of the fabrication processes will be explored, potentially allowing rapid, high-volume production of PHAs.

## Data Availability Statement

The raw data supporting the conclusions of this article will be made available by the authors, without undue reservation.

## Author Contributions

JC contributed to the design conception, experimental and experimental system design, measurement, data analysis, modeling, and manuscript preparation. MC contributed to device modeling, experimental design, and manuscript preparation and review. NG contributed to the design ideation and manuscript review. KO and PV contributed scientific support, coordination, layout, and revision support for the manuscript preparation. All authors contributed to the article and approved the submitted version.

## Conflict of Interest

The authors declare that the research was conducted in the absence of any commercial or financial relationships that could be construed as a potential conflict of interest.

## Supplementary Material

Supplementary Video 1 Parallel Helix Actuators: fabrication and individual chamber characterization.

Supplementary Video 2 Parallel Helix Actuators: 3D workspace evaluation under tip loading.
